# Overview of Primary Angiitis of the Central Nervous System: Current Insights

**DOI:** 10.26502/aimr.0236

**Published:** 2026-03-05

**Authors:** Amy Phu, Devendra K Agrawal

**Affiliations:** 1 Department of Translational Research, College of Osteopathic Medicine of the Pacific, Western University of Health Sciences, Pomona, California 91766 USA

**Keywords:** Circulating endothelial cells, Cyclophosphamide, Encephalopathy, Ischemic stroke, Neurofilament light chain (NfL), Neuroinflammation, Primary angiitis of the central nervous system (PACNS), Primary central nervous system vasculitis, Rituximab, TNF-α inhibitors

## Abstract

Primary angiitis of the central nervous system (PACNS), also known as primary central nervous system vasculitis, is a rare autoimmune condition characterized by exclusive involvement of the brain, spinal cord, and leptomeninges. With an estimated prevalence of 2.4 cases per million person-years in North America, PACNS represents one of the rarest vasculitides and continues to pose significant diagnostic and therapeutic challenges due to its heterogeneous clinical manifestations and lack of specific biomarkers. This critical review article provides a comprehensive overview of PACNS with emphasis on: (i) current understanding of pathophysiology and unique variations of clinical presentation, (ii) challenges and strategies in reaching a definitive diagnosis, and (iii) evaluation and comparison of pharmacological and neuromodulatory therapies currently available. Recent advances in understanding pathophysiology have identified elevated interleukin-17 in cerebrospinal fluid, distinct gene expression profiles including upregulation of TBC1D3C and TBC1D3L genes, and immune cell profiling showing increased intrathecal NK-cell and B-cell frequencies. Three histopathological subtypes have been characterized: granulomatous vasculitis (32–61%), lymphocytic vasculitis (24–79%), and necrotizing vasculitis (14–42%). Emerging biomarkers including neurofilament light chain, circulating endothelial cells, and soluble triggering receptor expressed on myeloid cells 2 (sTREM2) show promise for diagnosis and disease monitoring. Advanced neuroimaging modalities, particularly high-resolution vessel wall magnetic resonance imaging and positron emission tomography, demonstrate utility in detecting vessel wall inflammation and guiding biopsy site selection. Treatment approaches include glucocorticoids combined with cyclophosphamide for induction therapy, with mycophenolate mofetil, rituximab, azathioprine, and methotrexate used for maintenance. TNF-α inhibitors have shown efficacy in refractory cases. Despite treatment, mortality ranges from 8–23%, and approximately 25% of patients experience severe disability. PACNS remains a diagnostically challenging condition requiring high clinical suspicion and comprehensive workup. Recent advances in understanding immunopathological mechanisms, identification of novel biomarkers, and development of advanced neuroimaging techniques offer promise for earlier diagnosis and more targeted therapeutic interventions. Brain biopsy remains the gold standard for diagnosis, though emerging non-invasive diagnostic modalities may reduce reliance on invasive procedures. Treatment strategies continue to evolve beyond traditional glucocorticoid and cyclophosphamide regimens, with biologics including rituximab and TNF-α inhibitors showing efficacy in refractory disease. Future multicenter collaborative studies are essential to validate emerging biomarkers, refine diagnostic criteria, establish evidence-based treatment protocols, and improve outcomes for this rare and heterogeneous disorder.

## Introduction

Primary central nervous system (CNS) vasculitis, or synonymously, primary angiitis of the central nervous system (PACNS), is a rare autoimmune condition defined by exclusive involvement of the brain, spinal cord, and leptomeninges. PACNS was not formally recognized as its own clinical entity until the 1950s [1,2]. Comparatively, secondary angiitis of the central nervous system (SACNS) typically occurs as a late manifestation of another systemic disorder [3]. This review will focus on PACNS, one of the rarest vasculitides that continues to pose a significant challenge for diagnosis and treatment due to its complex and highly unspecific clinical manifestations.

Currently, there are no clearly established risk factors identified within the medical literature and further research is needed to gain a better understanding of the patient population and potential genetic and environmental factors that may possibly lead to a patient developing PACNS. However, certain hypotheses have been proposed, including infectious triggers, immunopathogenic mechanisms, and cerebral amyloid angiopathy as potential contributors to disease pathogenesis [5]. Compared to secondary CNS vasculitis which occur concomitantly with systemic diseases, connective tissue disorders, and infections, there are no predisposing conditions associated with PACNS.

Unlike systemic vasculitis, our understanding of PACNS pathogenesis and immunopathological mechanisms are currently limited; its estimated prevalence of 2.4 million cases per million person-year in North America makes it among the rarest of vasculitides [4]. It has the propensity to affect patients of all ages, although it affects mainly middle-aged individuals, with a median age of onset of 50 and a similar prevalence between male and female patients [1]. However, some studies do report PACNS being twice as prevalent in men compared to women The mortality rate is reported to be between 8–23%, and about 25% of patients suffer from severe disability despite treatment [1].

Cardinal nonspecific neurological clinical manifestations include headache, altered cognition, and focal neurological deficits such as visual disturbances, dysarthria, aphasia, ataxia, and hemiparesis [4]. High clinical suspicion for PACNS and comprehensive workup should be performed for patients with nonspecific neurological complaints. Systemic vasculitides typically present with constitutional symptoms, elevated inflammatory markers, and involvement of multiple organs, while PACNS is characterized by its neurological clinical picture in the absence of systemic symptoms [3].

In this article, we critically reviewed the literature in the last two decades with primary emphasis on clinical case reports and original research articles to provide an overview of the following points regarding PACNS: (i) current understanding of its pathophysiology and unique variations of clinical presentation, (ii) challenges and strategies in reaching a definitive diagnosis, and (iii) evaluation and comparison of pharmacological and neuromodulatory therapies currently available.

## Methods

A review of literature was conducted encompassing data from September 1959 through January 2026, with primary focus on case reports published from 2010 to 2026. A literature search was performed in PubMed using keywords including “primary angiitis of the central nervous system,” “primary central nervous system vasculitis,” “immunological mechanisms,” “infliximab”. Of 124 articles initially reviewed, 72 were selected for inclusion based on relevance, quality, and English language. PACNS is divided into two main subtypes: small-vessel and medium-to-large vessel disease, each with distinct diagnostic methods and clinical outcomes.

### Pathophysiology of Primary CNS Vasculitis

**Figure 1:**
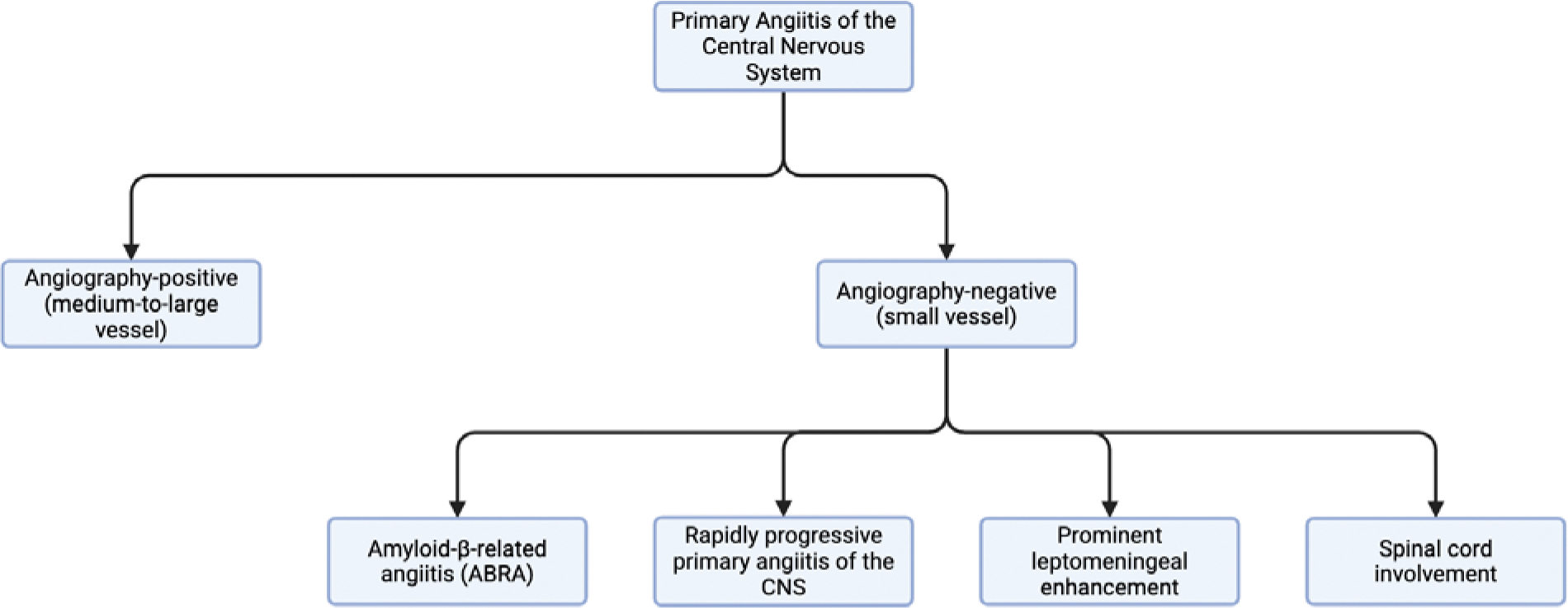
Flowchart detailing subtypes of PACNS currently recognized in the literature.

Although the pathophysiology of primary CNS vasculitis (PCNSV) continues to be poorly understood, several key molecular mechanisms and immune pathways contributing to disease pathogenesis have been identified in recent research. From a clinical standpoint, PACNS is divided into two main subtypes: small-vessel and medium-to-large vessel disease, each possessing their own respective diagnostic methods and clinical outcomes [1] (Figure 1). The proximal segments and secondary branches of cerebral arteries are typically classified as large/medium-sized vessels [6]. PACNS commonly presents with nonspecific neurological clinical manifestations such as headache, progressive cognitive impairment, or seizures. As the disease progresses, vascular inflammation may lead to vessel occlusion or rupture, with subsequent ischemic or hemorrhagic infarctions [1].

Research pertaining to pathophysiological mechanisms and immunopathology of PACNS shows a primary focus on cerebrospinal fluid (CSF) markers. Although no PACNS-specific immune signatures have been found, immune cell profiling of CSF in some patients showed increased intrathecal NK-cell or B-cell frequencies, with one third of patients exhibiting intrathecal Ig synthesis and antibody-secreting plasma cells in the CSF, demonstrating evidence of inflammatory processes within the brain [10].

Compared to patients with stroke not attributable to vasculitis or other noninflammatory neurologic disorders, elevated CSF levels of the proinflammatory cytokine interleukin-17 (IL-17) are found in patients with PACNS and SACNS during periods of both active disease and remission [8]. Furthermore, there is no significant difference in the production of interferon gamma (IFN-γ) in the CSF and no overall differences in the relative frequencies of peripheral immune cells [8]. Proinflammatory cytokines such as IFN-γ and IL-17 are potent mediators of chemokine release, activation of endothelial surfaces, and recruitment of inflammatory cells [8]. Specifically, IL-17 is produced mainly by CD4+ T cells, natural killer cells, and B cells [1]. It is involved in activation of dendritic cell maturation and antigen processing alongside neuroinflammation and inhibition of oxidative phosphorylation [7]. Thus, there lies potential in utilizing intrathecal IL-17 producing CD4+ T cells as a biomarker to aid in establishing the diagnosis of cerebral vasculitis in patients with ischemic stroke.

Recent studies reviewing cerebral biopsies derived from patients with PACNS from the Mayo Clinic describe it as a heterogenous condition with distinct histopathological patterns and clinical subsets with differing outcomes and responses to treatment [7].

A single case report describing the immunological phenotype of the cells comprising the inflammatory infiltrate in PCNSV revealed predominant infiltration by CD45R0+ T cells both in and around small vessels, supporting the role of memory T cells in pathogenesis of vasculitis [9].

Past studies have found that PACNS has a different transcription profile compared to normal brain tissue. Namely, the two top upregulated genes associated with PACNS were TBC1D3C and TBC1D3L, overexpressed in GV and ABRA subtypes. Both genes are guanosine triphosphatase which has the capability to activate Ras analog in brain 5 (RAB5) protein, which is an early endosome marker that regulates endocytosis and the recycling of membrane receptors [11]. Given that endosomal dysfunction and RAB5 activation has been reported in Alzheimer disease, Parkinson disease, amyotrophic lateral sclerosis, and other neurodegenerative disorders, some authors have speculated on RAB5 hyperactivation and changes in endosome trafficking involvement in PACNS pathogenesis, particularly in the GV and ABRA types [7,12].

Gene expression profiling has revealed granulomatous vasculitis and beta and a subgroup of patients with GV associated with cerebral amyloid angiopathy, amyloid beta related angiitis (ABRA) correlated with gene signatures associated with CD4+ naive T cells and monocytes [1]. Lymphocytic vasculitis demonstrates a higher expression of genes encoding constant chains of immunoglobulins, consistent with earlier findings showing occurrence of plasma cell signatures and γδ T cell involvement in the CNS of the lymphocytic subset of PACNS [1,7,13].

Although there are currently no universally recognized genes specific to PCNSV, recently there have been two PCNSV cases discussed in the literature describing presence of a partial recombination-activating gene 2 (RAG2) deficiency, which can present in patients with a variety of autoimmune-related conditions [14,15]. The RAG2 gene works with RAG1 to encode Recombination Activating Gene proteins which hold a crucial role in immunity by allowing recombination of genes at the T cell receptors to generate both T cells and B cells [15]. Further research is required to support the contribution of partial RAG2 deficiency in the pathogenesis of PCNSV.

Additionally, a case in the literature describes a PCNSV patient positive for HLA-B51 and A26. The patient experienced disease progression despite glucocorticoid therapy but had a good clinical response to infliximab [16]. HLA-B51 has been recognized as a contributor to the disease phenotypes of Behcet’s syndrome, with reports of 50–80% frequency of the gene in patients with Behcet’s syndrome in endemic geographies such as the Mediterranean basin, Middle Eastern and Far East Asian countries [17]. HLA-A26 has been found to have significant association with uveitis and gastrointestinal involvement in patients with Behcet’s disease [18]. Further research and greater understanding of the pathogenetic and molecular mechanisms implicated in PACNS may lead to new diagnostic and therapeutic strategies better tailored to PACNS as a whole and its individual subsets.

### Diagnosis of CNS Vasculitis

In the early decades of the 20th century, CNS vasculitis evaded establishment as its own clinical entity and was often erroneously classified at postmortem examination as granulomatous angiitis. In 1959, PACNS was at last defined as a separate clinical entity, differentiated by multinucleated giant and epithelioid cells on pathology [2]. For the next twenty years, patients with a diverse range of systemic and neurological disorders will be identified to have this disorder. However, there existed no effective treatments at the time, with early reports resulting in fatality and diagnosis confirmed by autopsy [1,2].

In 1988, diagnostic criteria for PACNS were initially proposed by Calabrese and Mallek which included [21]:

1. Unexplained neurologic deficit after thorough clinical and laboratory evaluation.
2. Arteritic process demonstrated by cerebral angiogram and/or histopathologic examination in CNS
3. Absent evidence of systemic vasculitis

However, these criteria were not validated in prospective studies, and there continues to be an absence of validated criteria and diagnostic guidelines for PACNS.

Thus, Birnbaum and Hellmann suggested revision of these criteria in 2009 with the intent of differentiating PACNS from reversible cerebral vasoconstriction syndrome (RCVS) using two subdivisions for level of certainty of diagnosis: “definite” vs “probable” [22]. Patients received a “definite” diagnosis of PACNS if there is biopsy confirmation of vasculitis, and probable diagnosis if tissue confirmation is not performed and there are high probability findings on angiogram in conjunction with abnormal findings on MRI and CSF profile consistent with PACNS. PACNS typically follows a relapsing-remitting course with undulating levels of disease activity warranting long term, continual monitoring [22].

Extensive laboratory testing and workup including gram staining, culture, serologic and molecular tests, cytologic analysis, flow cytometry, or detection of clonal rearrangements with a standard polymerase-chain reaction assay are essential in ruling out clinical and infectious mimickers. Given the lack of established diagnostic criteria and the low specificity of its diagnostic tools, PACNS is mainly a diagnosis of exclusion as it shares overlapping features with many other pathologies.

Serological markers of inflammation such as erythrocyte sedimentation rate and C-reactive protein are normal in most patients [22]. CSF is abnormal in approximately 75% of patients, characterized by a mildly increased leukocyte count (>5 cells per millimeter), increased protein concentration (>45 mg per deciliter) or both [1].

Furthermore, there is arising evidence of biomarkers in serum and CSF that may be supportive of both diagnosis and assessment of PACNS disease activity. This would provide a more cost effective and less invasive means of determining the appropriate clinical decisions to pursue, in addition to contributing further certainty in the diagnosis. A recent study has shown patients with active PACNS have significantly elevated levels of neurofilament light chain (NfL) in both serum and CSF relative to PACNS patients in remission and healthy controls, indicating significant neuroaxonal injury secondary to acute inflammation and ischemia [19].

NfL is one of four subunits of neurofilaments, which are highly specific major structural proteins of neuron cytoskeletons found to be highly sensitive for detecting neuroaxonal damage [19,20]. NfL has been recognized as a promising biomarker in a wide variety of neurological disorders such as multiple sclerosis, amyotrophic lateral sclerosis, and traumatic brain injury [19]. Notably, the authors recognize that although their findings reflect a correlation between CSF and serum NfL levels consistent with prior studies, the utility of serum NfL is limited at this time due to its significantly lower sensitivity compared to CSF. Furthermore, recent ischemic events as well as age-related elevations in baseline NfL are factors to consider with NfL-guided surveillance. The limitations of such studies are attributable to small sample sizes, highlighting the importance of future multicenter collaborations to validate these findings and their accuracy.

NfL continues to have limited specificity, especially when differentiating inflammatory from noninflammatory etiologies. Thus, integration of additional biomarkers such as circulating endothelial cells (CECs) may be helpful in overcoming this limitation and increasing diagnostic accuracy. [23]

CECs are an established sensitive and specific marker of endothelial injury, found to be increased in active vasculitis patients related to healthy controls and patients treated successfully with immunosuppressive treatments [24,25]. The underlying pathophysiology of CECs appears to involve detachment of endothelial cells from the vessel wall from either mechanical injury, inflammatory or non-inflammatory endothelial damage [26]. A recent study showed patients with active PACNS with significantly higher CEC levels in the peripheral blood than both PACNS patients in remission and healthy subjects. Additionally, compared to patients with reversible cerebral vasoconstrictive syndrome (RCVS) or Moyamoya disease (MMD), CECs were also significantly elevated compared to healthy controls, but still significantly lower compared to patients with active PACNS. Patients in remission from PACNS were found to have lower CEC values than patients with RCVS/MMD. Finally, this same study observed no difference between CECs in healthy individuals and patients in remission [24].

Lastly, soluble triggering receptor expressed on myeloid cells 2 (sTREM2) has been found to play a role for TREM2 in the pathogenesis of PACNS, based on abnormal increases in TREM2 in PACNS patients. Higher sTREM2 levels resulted in increased production of inflammatory factors, which leads to a larger lesion in the CNS and more severe clinical symptoms in PACNS patients. Higher sTREM2 levels were seen in PACNS patients with poor outcomes, hypothesized to be due to weakening of microglial functions including tissue repair, phagocytosis of dying cells, and control of local inflammation. This study showed a positive correlation between sTREM2 levels in PACNS patients and levels of pro-inflammatory cytokines (TNF-α, IL-6, IL-8, IL-1β) and complement (C4) [33].

Activation of microglia increases generation of cytokines, chemokines, and matrix metalloproteinases (MMPs), resulting in destruction of the blood-brain barrier (BBB) and allows cytokines from blood, complement components, and other immune cells to enter the CNS. Subsequently, this leads to further activation from microglia and a continued cascade of cytokine and complement increases, promoting neuronal injury. Simultaneously, macrophages in peripheral blood secrete high levels of blood-derived inflammatory factors passing through the damaged BBB into the CNS, promoting CNS inflammation [34]. In summary, sTREM2 serum and CSF levels may potentially be valuable biomarkers in monitoring disease severity, predicting lesion volume, and predicting prognosis in patients with PACNS [33].

Although these are promising findings, the validity of the results and the reliability as biomarkers for detecting active PACNS and differentiating it from similarly presenting disorders such as RCVS and MMD remains limited due to the small sample size of the study limited by single center. Additional observational studies are required, ideally in larger cohorts of active, biopsy confirmed vasculitis patients to establish definite conclusions.

In certain cases, imaging, cerebrospinal fluid evaluation, serologic, and even angiography can fail to establish a diagnosis, requiring a brain biopsy [14,27]. The gold standard for diagnosis remains brain parenchymal and meningeal biopsy with histopathological classification [14,30]. A wedge-shaped sample from the temporal lobe of the non-dominant side in areas with longitudinally arranged surface vessels is the preferred site for brain and leptomeningeal biopsy [28]. In cases where open biopsy is not a viable option, stereotactic biopsy guided by MRI has been used to diagnose small vessel PACNS, targeting the areas with active inflammation documented by increased MET uptake on PET to reduce the risk of sample error [29]. Such stereotactic techniques to obtain brain biopsy are less traumatic and better for deep seated brain lesions compared to open biopsy; however, more research is required to further refine the diagnostic process PACNS. Additionally, given the segmental nature of the disease, biopsies may show falsely negative results, adding a further element of complexity to this challenging diagnosis [28]. The chance of false negative results is reduced by biopsying radiologically abnormal tissue and sampling both meningeal and cortical tissues [30].

The wide variation in histopathological findings and recent reports suggest that PACNS is more likely to be a spectrum of disorders rather than an isolated clinicopathological entity. The three subtypes that have been identified on tissue biopsy include: granulomatous vasculitis (32–61% of patients), lymphocytic vasculitis (24–79% of patients), and necrotizing vasculitis (14–42%). These usually remain stable over time in an individual patient, but at times may overlap. Granulomatous vasculitis present with mononuclear inflammation with well-formed granulomas and multinucleated giant cells in vessel walls and may be associated with amyloid-beta vascular deposition in Aβ-related angiitis. Lymphocytic vasculitis is characterized by lymphocyte infiltration in the absence of granulomas. Necrotizing vasculitis is less common and presents histologically similarly to polyarteritis nodosa, characterized by transmural fibrinoid necrosis. Necrotizing vasculitis has been found to be linked to intracranial hemorrhage, whereas lymphocytic vasculitis might have a milder disease course characterized by lower rates of disability and mortality [1].

A review of the literature in 2026 by Rai et al. discovered a total of 49 reported cases of unilateral PACNS (U-PACNS). PACNS is typically bilateral in presentation; thus, the rare unilateral variant tends to be underrecognized and misdiagnosed as glioma, demyelination, or chronic encephalitis given its similar clinical and imaging features. The manifestations of U-PACNS include tumor-like solitary or multifocal lesions, hemorrhages, or infarcts alongside nonspecific imaging findings and small-to-medium-vessel lymphocytic vasculitis. Bilateral disease is more commonly associated with granulomatous inflammation [34].

Typical histopathological findings of brain biopsy from PACNS patients include fibrinoid changes in small muscular blood vessels and predominantly lymphocytic perivascular inflammatory infiltrate with occasional histiocytes and multinucleated giant cells [36]. Other typical findings on brain biopsy include intramural mononuclear lymphocytic inflammation of small and medium sized vessels in gray and white matter [37]. Other reported cases describe transmural inflammation affecting all vascular layers, leading to thrombosis, necrosis, and macrophagic infiltration, closely mimicking glioblastoma [38]. An even rarer finding includes marked perivascular infiltration of histiocytes with sheets of xanthomatous cells [39].

About a quarter of patients with biopsy-positive primary CNS vasculitis have evidence of cerebral amyloid angiopathy (CAA) [41]. CAA is characterized by vascular deposits of beta amyloid, in addition to granulomatous vasculitis; PACNS patients with biopsies possessing these features are classified as having amyloid beta-related angiitis (ABRA) [1,36,40]. ABRA may also be found in association with eosinophilic meningitis, an exceedingly rare occurrence with only a few cases published in the medical literature.

Since increased levels of eosinophils are typically not observed in the peripheral blood or CSF in patients with eosinophilic CNS vasculitis, the only way to diagnose it is via brain biopsy. The common histopathological findings are predominantly eosinophilic infiltrates of medium-sized leptomeningeal arteries without granulomas or giant cells, in addition to cortex and vessel walls without beta amyloid [41]. However, some cases do report findings of numerous beta-amyloid depositions in the cortex and vessel walls in addition to vasculitis with granulomas and giant cells [42].

The limitations of using imaging in diagnosing CNS vasculitis lie in its propensity to affect vessels of all sizes. While medium and large vessel inflammation is easily visualized on conventional imaging techniques and HR-VWI, inflammation of small arteries and leptomeningeal vessels is difficult to determine using conventional imaging techniques [43] (Table 1).

Traditional techniques such as cerebral angiography and MRI still hold diagnostic value and should be included in primary work-up to exclude clinical mimickers. Importantly, MRI of the brain is abnormal in more than 90% of patients; thus, an unremarkable MRI suggests PACNS is highly unlikely [22]. MRI typically shows multifocal cerebral lesions in both gray and white matter with enhancement in T2 and fluid attenuated inversion recovery (FLAIR) images without mass effect or tumor-like lesions, while conventional angiography and diffusion-weighted images are often normal [27,44]. Comparatively, digital subtraction angiography (DSA) holds high sensitivity in detecting vasculitis, especially for small-vessel and medium-vessel vasculitis [14] (Table 1).

Although most cases of PACNS reported in the literature present without mass effect or tumor-like lesions on MRI, clinicians must maintain hypervigilance and caution in diagnosis and management of patients presenting with imaging suggestive of CNS tumors and malignancy. A recent systematic review of published cases from 2003–2023 found 34 tumor-like PACNS cases with similar characteristics to glioblastoma [31]. Diffusion-weighted MR imaging has been shown to aid in detection of such lesions; typical findings show hyperintense lesions with heterogeneous average diffusion coefficient (ADC) values, indicative of coexistence of vasogenic and cytotoxic edema [14,31,45]. Additionally, there exists in the literature an unusual case of an entity resembling a malignant tumor on sinus CT and skull base MRI located on the clivus that was subsequently diagnosed via histopathology as PACNS, illustrating that while bone destruction is typically considered a manifestation of malignancy, mass PACNS is also capable of bone destruction [50]. Histopathological confirmation via tissue biopsy in patients presenting with nonspecific neurological symptoms is essential in confirming diagnosis of PACNS; misdiagnosis may lead to delays in proper treatment and result in devastating neurological sequelae and even death.

The use of whole body 18-fluorodeoxyglucose PET combined with computed tomography can help support small vessel PACNS (svPACNS) diagnosis through exclusion of inflammation present outside the CNS [32,46]. Furthermore, given the segmental nature of the disease, brain biopsy may yield false negative results. Two biopsy proven cases of small vessel PACNS showed both brain FDG- and MET-PET can detect high metabolic activity beyond brain MRI abnormalities, suggesting they might have utility in identifying regions of the brain with active inflammation thus improving spatial targeting of brain biopsy. Further research is required to confirm the consistency of these findings, but there is promising evidence that FDG- and MET-PET should be systematically performed when svPACNS is suspected [46].

In recent decades, the advent of advanced neuroimaging modalities such as high-resolution vessel wall magnetic resonance imaging (HR-VWI) provide additional non-invasive means of directly visualizing vessel wall inflammation and thickening in patients with intracranial arterial vasculopathies. The most common feature supporting imaging supported diagnoses of primary CNS vasculitis utilizing HR-VWI is intense, concentric vessel wall enhancement, particularly of the right middle cerebral artery (M1) wall and the anterior circulation [49]. Similar findings were found in a small sample of patients with infectious CNS vasculitis; thus HR-VWI may have limited utility as a tool in differentiating primary CNS vasculitis from secondary CNS vasculitis.

Comparatively, secondary CNS vasculitis had strong correlations with arterial stenosis of both anterior and posterior circulation in time of flight (TOF) images and chronic white matter lesions in FLAIR images [47]. Authors have found evidence enhancement could be directly associated with disease activity and thus have utility in monitoring treatment response and disease progression [48,49]. Although HR-VWI-MRI appears to be a promising technique with diagnostic value, further research involving multicenter studies is required to confirm its validity.

### Clinical Presentation of Primary Angiitis of CNS

The highly variable clinical presentation of PACNS makes the formation of a universal set of symptoms acceptable for use in diagnostic guidelines extremely challenging. Clinical onset of PACNS is commonly subacute, characterized by a gradual, insidious progression of symptoms [51]. However, it can also present with acute onset and rapidly progress within a few days or weeks. The most frequent findings at initial presentation are headache and cognitive impairment, followed by stroke and visual symptoms [54].

Additionally, although PACNS primarily affects adults, clinicians must remain hypervigilant for potential PACNS in young children and adolescents. A 13-year-old patient presenting with atypical findings of epilepsy and headache with nonspecific imaging findings evaded accurate diagnosis of U-PACNS and treatment for 3.5 years, underscoring the importance of histological analysis in patients with neurological symptoms refractory to other treatments [55]. Refractory status epilepticus in PACNS patients may occur due to both ischemic and hemorrhagic stroke [61]. Another report describes a 5-year-old patient presenting to the emergency department with new-onset status epilepticus and fever, uniquely complicated with elevated intracranial pressure requiring decompressive hemicraniectomy with favorable neurologic outcomes [58].

A 2016 study by Boysson et al. found that patients with isolated small-vessel PACNS are typically younger and more commonly present with seizures, cognitive impairment, altered consciousness and dyskinesias, resembling progressive encephalopathy rather than a stroke-like presentation [56,57]. The rise of certain clinical patterns attributable to subsets of PACNS in recent times will hopefully reduce time between onset, diagnosis, and treatment. Although uncommon, PACNS patients may present with progressive leukoencephalopathy, the clinical presentation resembling lymphoproliferative or infiltrative disease processes such as sarcoidosis, neoplastic, or histiocytic conditions such as akinetic mutism and shuffling gait. Imaging findings may include a pattern of white matter hyperintensity, prompting initial concern for lymphoma; however, biopsy revealed lymphocytic infiltration of the small vessels characteristic of vasculitis [58].

A large cohort study including clinical and imaging profile, and long follow up data including treatment found an association between elevated baseline stroke severity and poor long-term outcomes, while an abnormal angiogram was often seen in those with good outcomes; thus, the authors recommend that in patients with a strong suspicion of small vessel PACNS, more invasive diagnostic tests should be considered even in the presence of a negative angiogram [60]. This same study supported previous findings that the medium/large vessel vasculitis subtype often presented as a stroke, compared to small vessel vasculitis subtype which often had an inflammatory CSF with greater delays in diagnosis attributed to its subacute presentation [61]. Given the high potential for morbidity and mortality with misdiagnosis and delays in treatment in PACNS, biopsy should be pursued as a viable option. Since PACNS is a relatively recently defined clinical entity, hopefully we can continue ascertaining and gain a deeper understanding of each phenotypic presentation as more cases are reported and reviewed in the medical literature.

Another rare complication of CNS vasculitis is hydrocephalus, with only two known cases reported in the literature to date [52]. Despite initially responding well to corticosteroid therapy, both patients developed signs and symptoms of hydrocephalus including imbalanced gait and urinary incontinence; thus, on clinical worsening of patients with PACNS, the possibility of hydrocephalus should be considered [52,53].

Further contributing to the kaleidoscopic nature of PACNS symptomatology, this disorder may also rarely present with spinal symptoms like those found in multiple sclerosis (MS). As previously mentioned, the likelihood of the diagnosis being PACNS is significantly lower given a normal brain MRI. In one case, the patient presented with clinical manifestations typical of MS in the context of normal brain MRI; namely, progressive paraparesis and dysesthesia; notably, several cases report PACNS patients developing brain lesions later despite an initial clinical presentation of purely spinal symptoms [61]. Long term follow-up and management are essential in maintaining optimal therapeutic outcomes in these complicated cases.

In one case, the patient presented with a four-year history of dizziness and bladder dysfunction along with weakness in both legs with a slow and progressive gait dysfunction, with both clinical and radiological features suggestive of MS. This patient was treated with beta-interferon followed by worsening of clinical presentation. Thus, this case highlights the need for suspicion of vasculitis in the presence of micro/macrobleeds in conjunction with persistent contrast enhancing lesions [63]. Thorough diagnostic evaluation, including vascular imaging and histological verification, ideally via stereotactic biopsy, is essential.

Another unusual presenting finding of PACNS is fever and leukocytosis [64]. A patient presenting with a headache, blurred vision, mild fever (37.6°C) and leukocytosis with diffusion weighted imaging (DWI) showing multiple punctate hemorrhages in necrotic rim-enhancing lesions was diagnosed with unusual brain abscess and initiated on antibiotic treatment with no clinical improvement [65]. However, a retrospective review of MRIs revealed the presence of prominent microbleeds in the necrotic rim-enhancing lesions and heterogeneity of the DWIs suggested tumefactive PACNS rather than brain abscess. This case illustrates the need for a multidisciplinary, team-based approach for PACNS patients since there may be a significant discrepancy between clinical symptoms and imaging findings.

### Treatment of Primary Angiitis of CNS

The management of primary angiitis of the central nervous system (PACNS) remains challenging due to the lack of a universally accepted, evidence-based clinical guideline for treatment. Other therapeutic treatment strategies are based on retrospective studies and observational cohort studies, not randomized controlled trials, with treatment approaches based on systemic vasculitis [66]. Despite the limitations, early initiation of immunosuppressive therapy can improve neurological outcomes and reduce relapse [5].

For most patients with mild disease, characterized by isolated headaches and minimal neurological deficits and imaging, empiric treatment is high-dose glucocorticoid monotherapy or in combination with cyclophosphamide. Typically, the initial dose is 1 mg/kg of oral prednisone. For patients with moderate to severe disease, defined by encephalopathy, stroke, seizures, rapidly progressive deficits, or angiographic evidence of large-vessel involvement, induction therapy consists of intravenous high dose glucocorticoid (1 g/ day for 3–5 days), followed by an oral taper. There has been no evidence of initiation with oral or IV is superior to the other. In cases with severe neurological deficits or progressive disease, IV cyclophosphamide (800–1000 mg/month for 6 months) is added to glucocorticoid therapy to achieve disease control and reduce relapse risk [4,5,67]. Additionally, combination therapy is associated with lower relapse rates in patients with large-vessel involvement [4]. Infliximab and Etanercept or Rituxamab can be used for refractory PACNS [67,68]. TNF-alpha inhibitors and mycophenolic acid derivatives have shown success in treating patients who have resistance to both glucocorticoid and immunosuppressants [5].

After induction of remission, patients are transitioned to maintenance therapy with azathioprine, methotrexate, rituximab or mycophenolate mofetil for 12–18 months [3,4]. These agents are favored due to their use in systemic vasculitis and PACNS cohort studies. However, methotrexate is not the preferred maintenance agent as it does not penetrate the blood brain barrier significantly [1]. The choice of the agent is individualized and based on treatment response, comorbid conditions, and patient tolerance since there are no direct studies on the effectiveness of the drug. Despite maintenance therapy, approximately 26% of patients will experience relapse, defined as the progression or recurrence of lesion on MRI [66]. This highlights the need for prolonged monitoring.

Monitoring treatment response can be done through a combination of repeat MRI, angiography, and CSF analysis [4,5,66]. Additionally, there have been new imaging modalities to monitor treatment responses. One case describes utilizing 2D high resolution vessel wall MRI (HR-VWI) to directly visualize vessel wall inflammation, with concentric enhancement correlating with disease activity and treatment response [49].

Individualized treatment tailored for the subsets of PACNS requires further investigation as well. Some patients continue to experience neurologic deterioration following cessation of corticosteroid treatment, suggesting failure of initial immunosuppressant treatment and indicating the need for stronger agents, longer treatment duration, or both [28]. All patients responded to long term treatment with corticosteroids combined with other cytotoxic immunosuppressive agents. There are reports of fewer relapses for adults with PACNS receiving corticosteroids and cyclophosphamide for 12 months (10%) relative to those receiving the same regimen for 6 months (30%) or corticosteroid monotherapy (90%). In one case, long term clinical and radiological stabilization was unable to be achieved on steroid sparing agents alone, requiring a combination of oral mycophenolate mofetil 1.5mg/kg per day and prednisolone 0.3mg/kg per day [28]. These cases of disease recurrence years after cessation of immunosuppressant therapy implies long term follow up is necessary in patients with PACNS as recurrence of disease is possible even after many years of clinical dormancy.

A recently reported case of PACNS describes a solitary lesion treated with resection alone, followed by an absence of recurrence without additional immunosuppressive therapy The authors note that lesions are frequently multifocal rather than solitary; however this case suggests selected cases of lymphocytic vasculitis may possibly be treated with resection alone, which is a novel therapeutic approach previously never reported [69]. However, the postoperative follow-up period was short, and further observations are required to definitively suggest surgical treatment alone is sufficient for long-term disease control.

There exist a few reports in the literature of PACNS patients experiencing ischemic stroke after receiving rituximab infusions, hypothesized to be due to infusion-related reactions [70]. Studies conducted in patients with subarachnoid hemorrhage have found that IL-6 plays an important role in contributing to occurrence of vasospasm in cerebral vasculature [71]. This case and its debilitating patient outcomes of hemiplegia in the patient suggests there is a need for caution in administering rituximab in patients with documented severe stenosis of intracranial arteries.

Furthermore, there are reports of patients experiencing transient ischemic attacks shortly after initiation of corticosteroid therapy, while other patients had further neurological deterioration after corticosteroid withdrawal, suggesting the need for both long-term follow up and the search for medications other than immunosuppressive agents [28].

There is currently limited information on imaging changes in PACNS patients over time. One study analyzing the time course of vessel wall enhancement in PACNS patients undergoing treatment with immunosuppressants found a decrease in vessel wall enhancement until approximately 1 year after treatment began, and approximately half of vessels reached zero enhancement; changes were more pronounced in younger patients than in older ones. Additionally, there was little variation in stenosis grading and significant reduction of stenosis after initiation of therapy was rarely observed [72].

## Conclusion

Primary angiitis of the central nervous system remains one of the most diagnostically and therapeutically challenging vasculitides, characterized by significant variation in clinical presentation, histopathology, and outcomes. Despite advances in understanding PACNS over the past two decades, substantial gaps persist in our knowledge of disease pathogenesis, optimal diagnostic approaches, and evidence-based treatment strategies. The rarity of PACNS, combined with its nonspecific clinical manifestations and numerous mimickers, continues to result in diagnostic delays that adversely impact patient outcomes.

## Figures and Tables

**Table 1:** Comparison of the major features of small vessel and medium-to-large vessel PACNS.

Small Vessel (angiography −)	Medium-to-Large Vessel (angiography +)
Cognitive dysfunction, tumor- like lesions and prominent meningeal/parenchymal enhancement on MRI	Acute cerebrovascular events, limb weakness, ischemic infarctions
Markedly elevated CSF protein levels	100% show vascular stenosis on angiography
Require brain biopsy for diagnosis	94% show circumferential vessel wall enhancement on high-resolution MRI
Median age of onset 30.5 years	Median age of onset 40.5–50 years
Relapse rate of 89%	Relapse rate of 30%
Median diagnostic delays of 620 days	Diagnosis without biopsy possible based on angiographic findings and vessel wall imaging
Glucocorticoids alone preferred, especially in patients without infarctions	Cyclophosphamide and glucocorticoids for patients with multiple infarcts

## Key Points

- Elevated interleukin-17 (IL-17) in CSF distinguishes PACNS from non-inflammatory neurologic disorders. Immune cell profiling shows increased intrathecal NK-cell and B-cell frequencies with evidence of intrathecal immunoglobulin synthesis. Gene expression profiling reveals upregulation of TBC1D3C and TBC1D3L genes, and distinct molecular signatures associated with different histopathological subtypes.
- Serological markers of inflammation, including ESR and CRP, are normal in most patients. CSF is abnormal in 75% of patients characterized by mild leukocytosis and/or increased protein concentration. In many cases, particularly in those of small-vessel PACNS, tissue biopsy is necessary to confirm diagnosis and avoid unnecessary immunosuppressive therapy.
- High-resolution vessel wall MRI (HR-VWI) can directly visualize vessel wall inflammation and enhancement, with evidence suggesting correlation with disease activity. FDG- and MET-PET imaging can identify regions of active inflammation beyond conventional MRI abnormalities, potentially improving spatial targeting of brain biopsies and reducing false-negative results.
- Three histopathologic subtypes of PACNS have been identified so far, each with varying prognosis: granulomatous vasculitis (32–61%, associated with worse outcomes and relapsing-remitting course), lymphocytic vasculitis (24–79%, milder disease course with lower disability and mortality), and necrotizing vasculitis (14–42%, associated with intracranial hemorrhage).
- PACNS typically affects bilateral hemispheres of the brain but given the heterogeneity of its clinical manifestation clinicians must be vigilant in suspecting PACNS patients presenting with unilateral lesions on MRI.
- Neurofilament light chain (NfL) levels in CSF and serum are significantly elevated in active PACNS compared to remission. Circulating endothelial cells (CECs) are elevated in active disease and significantly higher than in RCVS or moyamoya disease. Soluble TREM2 (sTREM2) correlates with disease severity and prognosis, though all require validation in larger multicenter studies.
- Brain biopsy remains the gold standard for diagnosis but has significant limitations, including false-negative results due to the segmental nature of the disease (sensitivity 30–50%). The preferred biopsy site is a wedge-shaped sample from the temporal lobe non-dominant hemisphere. Stereotactic MRI-guided biopsy targeting areas of increased metabolic activity on PET may improve diagnostic yield.
- Despite treatment, outcomes remain suboptimal with significant morbidity and mortality: Mortality ranges from 8–23%, and approximately 25% of patients suffer severe disability despite treatment. Only two-thirds of patients achieve good neurological outcomes. Granulomatous PACNS has the worst functional outcome (median mRS 4) and highest mortality (25%), with poor response to standard induction therapy (29% response rate). Poorer survival is associated with older age, longer diagnostic delay, and necrotizing subtypes.
